# Shifting narrative perspective and construal level shape emotional response and enhance eudaimonic well-being

**DOI:** 10.1038/s41598-025-01946-8

**Published:** 2025-06-05

**Authors:** Xuan Gu, Chi-Shing Tse, Ho-Chung Tsang, Taoran Zeng

**Affiliations:** 1 Department of Social Work, School of Philosophy and Social Development, Shandong University, Jinan, China; 2 Department of Educational Psychology, The Chinese University of Hong Kong, Hong Kong, China; 3 Centre for Learning Sciences and Technologies, The Chinese University of Hong Kong, Hong Kong, China

**Keywords:** Autobiographical memory, Construal level, Eudaimonic well-being, Narrative perspective, Positive and negative affect, Psychological distance, Psychology, Human behaviour

## Abstract

Understanding how people process and reflect upon past negative experiences is critical for promoting eudaimonic well-being. This study examined how psychological distance and construal level during autobiographical memory retrieval jointly influence post-recall affect and eudaimonic well-being. Participants retrieved negative autobiographical events initially from a 1st-person perspective and subsequently, on a separate day, from a 3rd-person perspective, thereby increasing psychological distance. During each retrieval, participants elaborated either at a high construal level (reflecting on the event’s broader meaning and life implications) or a low construal level (focusing on contextual details, such as location). After each retrieval, participants evaluated mnemonic characteristics of the recalled events, post-recall affective responses, and current eudaimonic well-being. Baseline measures included depression symptoms, dispositional construal level, and initial eudaimonic well-being. Results showed that high-level construal, compared to low-level construal, enhanced subjective vitality and search for meaning in life, while reduced negative post-recall affect. Shifting from a 1st-person to a 3rd-person perspective decreased emotional intensity and further increased meaning-seeking. Notably, beneficial effects of high construal level on eudaimonic well-being were more pronounced when recalling from the 1st-person (vs. 3rd-person) perspective. These findings offer new insights into Construal Level Theory and offer practical implications for expressive writing and well-being intervention.

**Supplementary Information:**

The online version contains supplementary material available at 10.1038/s41598-025-01946-8.

Autobiographical memory (AM)—the recollection of personal experience from one’s past—plays a critical role in shaping identity, regulating emotion, and supporting well-being^1-3^. The present study introduces a novel experimental paradigm designed to manipulate orthogonally two key factors during AM retrieval: construal level (focusing on contextual details vs. deriving personal meaning) and psychological distance (narrating memories from a 1st-person vs. 3rd-person perspective). Our primary research questions are to examine how these factors interact to influence post-recall affect and eudaimonic well-being in young adults following the recall of negative AM events. Theoretically, investigating the combined effects of construal level and psychological distance on AM retrieval offers new insights into memory reconstruction processes and extends Construal Level Theory by exploring its applicability in emotional memory retrieval. Practically, this paradigm may identify cognitive mechanisms underlying the emotional consequences of AM retrieval, thus informing the development of a high-construal, meaning-oriented intervention approach that harnesses AM retrieval to enhance mental health and well-being.

## Psychological distance and construal level

In the context of AM, psychological distance can be defined as an individual’s subjective perception of time and distance separating them from personally experienced past events^4,5^. According to Construal Level Theory^6,7^, behaviors—including AM retrieval—are represented at varying levels of abstraction, e.g., lower construal of writing an essay involves connecting words, whereas higher construal means expressing ideas to influence others. When retrieving AM events at a lower construal level, individuals pay more attention to contextual details, such as the persons involved, time, and location of the event. In contrast, retrieving AM events at a higher construal level involves reflecting on an its meaning, values, and broader personal implications. Construal Level Theory further proposes a link between psychological distance and construal level—psychologically distant events are represented at higher construal levels characterized by their abstract features, whereas psychologically proximal events are represented at lower construal levels characterized by concrete features.

However, some findings, despite not specifically about AM, have challenged this link between construal level and psychological distance^8-11^. For example, Williams, Stein, and Galguera^12^ manipulated construal level and psychological distance simultaneously, observing distinct or even contradictory effects on participants’ affect evaluation. Similarly, Gu and Tse^13^ examined construal level and psychological distance in relation to self-transcendence and self-enhancement values, which inherently represent conflicting motivational goals. Results showed that participants construed self-transcendence values at a higher, more abstract level than self-enhancement values. However, they also perceived strangers endorsing self-transcendence values as psychologically closer compared to those endorsing self-enhancement values^1^. These suggest that self-transcendence values, despite their more abstract construal, may be experienced as psychologically more closely than self-enhancement values, incongruent with the link between construal level and psychological distance postulated by Construal Level Theory.

Despite substantial evidence supporting the roles of construal level and psychological distance in various cognitive processes such as judgment and decision making, to our knowledge, no published study has directly examined their interactive effects in relation to AM retrieval, individuals’ perception of retrieved events (e.g., perceived emotional intensity), or potential implications for individuals’ eudaimonic well-being. Few studies have reported the beneficial effects of psychological distance manipulations—such as shifts in narrative perspective—on attenuating emotional intensity associated with negative AM events^4,14^. The current study aims to extend these works and test (a) how construal level (emphasizing either contextual details or meaning) and psychological distance (1st-person vs. 3rd-person narrative perspective) during AM retrieval influence individuals’ post-recall affect and eudaimonic well-being and (b) whether the effects of construal level and psychological distance would be interactive, which will have substantial implications on the Construal Level Theory.

### Autobiographical memory, construal level, and well-being

AM retrieval may modulate one’s mood^15^, facilitate coping with negative emotions^16^, and enhance well-being^17^. How AM retrieval is framed or construed can influence one’s subsequent well-being^18,19^. When individuals retrieve AM by emphasizing the meaning of past events rather than their contextual details, they report more positive emotions, increased self-understanding, and express a greater sense of purpose in life^20^. AM events’ meaning or contextual details reflect the high vs. low construal level at which people retrieve negative AM. However, no published work directly manipulated the construal level at AM retrieval and examined its effect on emotion and well-being. In the current study, we manipulated the construal level at AM retrieval directly by instructing participants to retrieve and narrate a negative AM event with an emphasis on its contextual details or meaning. The contextual details of the event refer to where the event took place, the time of its occurrence, the people or things involved in the event, and what participants and other people did. The meaning of the event refers to what lesson that participants draw from the event, what impact the event has had on them, and why it is an important event in their life. This was expected to lead participants to construe their AM events at a high or low level. For example, when they retrieve a big quarrel with parents at a high construal level, they focus on why the event occurred, e.g., debating a social issue and its implication in life, understanding themselves and their parents’ political ideologies. When retrieving the event at a low construal level, they focus on contextual details, such as emotional wordings used during the quarrel.

There are several ways to define well-being^21^. In this study, we focus on eudaimonic well-being, which emphasizes meaning and personal growth since we examine the influences of high construal levels on AM retrieval by having participants recalled the meaning and values of their AM events. Individuals with higher levels of eudaimonic well-being tend to report a stronger sense of life meaning and personal development, as well as develop the best aspects of oneself^22,23^. Eudaimonic well-being contrasts with hedonic well-being, which centers on pleasures derived from daily life, material possessions, and instant enjoyment^22^.

To sum up, current research explores innovative ways of manipulating and accounting for construal level in AM. Since individuals who retrieve AM events at a high construal level represent these memories with a focus on meaning and values, we expected them to report greater eudaimonic well-being than those who retrieve AM events at a low construal level.

### Autobiographical memory, psychological distance, and post-recall affect

Given the link between construal level and psychological distance, it is reasonable to consider that psychological distance would exert similar effects as construal level on eudaimonic well-being and post-recall affect, as reviewed above. In the current research, we manipulated psychological distance by instructing participants to recall negative AM events from a 1st-person, followed by a 3rd-person narrative perspective. Narrative perspective is the personal pronoun one adopts to recall and describe AM events, which is related to visual perspective^14^. Shifting from the 1st-person pronoun (I) to the 3rd-person pronoun (he/she) reduces the emotional intensity of negative AM events through the mediating role of increased psychological distance^14^. Psychological distancing involves portraying the events and the self with a self-distancing perspective^24^, thus the more distant a person feels toward a negative event (e.g., shifting to a 3rd-person perspective from a 1st-person perspective when recalling an event), the weaker emotion and more meaning he/she would feel when recalling it^4,25,26,27^. AM retrieval also regulates one’s emotional states^28^; for example, by perceiving a negative AM event as psychologically distant, individuals loosen the association between the event and the self, thereby lowering its impact on emotion^29,30^. Moreover, shifting from a 1st-person to a 3rd-person perspective, instead of the reverse direction of shifting perspective, alters the intensity of emotional reactions^31^. Thus, in our experiment, we manipulated psychological distance by instructing participants to recall and narrate negative AM events first from the 1st-person perspective and then from the 3rd-person perspective.

Although Construal Level Theory proposes that construal level and psychological distance are closely intertwined or in reciprocal causation, some argue that they are not entirely overlapping and may have disparate effects on emotion^32^. Previous research did not tease apart the influence of construal level and psychological distance during AM retrieval. For example, it remains unclear whether the shift from the 1st-person to 3rd-person perspective (i.e., an increase in psychological distance) prompted a meaning-based retrieval and thus lessened perceived emotional intensity. In the current research, we aimed to examine whether psychological distance interacts with construal level in influencing eudaimonic well-being and post-recall affect. To do so, we orthogonally manipulated those two variables during AM retrieval. For example, participants could elaborate on the contextual details of an event (i.e., low construal level) from the 3rd-person perspective, or the meaning of the event (i.e., high construal level) from the 1st-person perspective. This design allowed us to explore the independent and interactive effects of psychological distance and construal level on emotional and well-being outcomes following AM retrieval.

### Current research

The purpose of the current study was to assess the effects of construal level and narrative perspective during AM retrieval on perceived emotional intensity of the recalled event, post-recall affect, and eudaimonic well-being by orthogonally manipulating these two variables within an experiment. When manipulating the construal level, we did not include a blank control group in which participants would receive no instruction about the construal level to retrieve AM events. As participants differ in dispositional construal level, the interpretation of the blank control group may be ambiguous. In line with prior research that purposefully excluded a blank control group for this reason^45,46^, we instead created an experimental condition to manipulate the construal levels during AM retrieval in two ways. Specifically, construal level was manipulated through instructions that were either embedded in the AM retrieval task or not directly related to the retrieval task. We refer to this as the relatedness of construal level manipulation, or manipulation relatedness (see below for the details of this manipulation).

Based on theoretical frameworks and prior findings, we had the following hypotheses.

- We expected that retrieving AM events at a high construal level, compared with a low construal level, would decrease perceived emotional intensity of the recalled event, reduce post-recall negative affect, and enhance eudaimonic well-being—main effect of construal level.
- We predicted that retrieving AM events from the 3rd-person perspective would trigger lower perceived emotional intensity of the recalled event (as shown in a previous study^14^, so this is a priori prediction), greater eudaimonic well-being, and lower post-recall negative affect than doing that from the 1st-person perspective—main effect of narrative perspective.
- There would be a construal level × narrative perspective interaction on perceived emotional intensity of the recalled event, eudaimonic well-being, and post-recall negative affect. The Construal Level Theory might predict that the effect of construal level is stronger for events retrieved from the 3rd-person (vs. 1st-person) perspective, supporting the conceptual link between construal level and psychological distance (as manipulated by narrative perspective). Alternatively, the narrative perspective × construal level interaction might show an opposite pattern—the effect of construal level would be stronger for AM events being retrieved from the 1st-person perspective (i.e., with a shorter psychological distance). As the emotional intensity of AM events with a longer psychological distance (i.e., those being narrated from the 3rd-person perspective) might be minimal, it would remain at a low degree regardless of the changes in construal level.
- There would be a manipulation-relatedness × narrative perspective × construal level interaction, or/and a manipulation-relatedness × construal level interaction on perceived emotional intensity of the recalled event, post-recall negative affect, and eudaimonic well-being. The narrative perspective × construal level interaction or/and the effect of construal level would be stronger when the construal level manipulation is embedded in AM retrieval than when it is not.

## Methods

### Sample estimation

We considered the partial eta-square reported in Gu and Tse^14^ about the effect of shifting narrative perspective from 1st person to 3rd person on reducing the perceived emotional intensity of negative AM events (0.16) and converted it to effect size *f* (0.44). In addition, the effect size reported in a meta-analysis for the effect of psychological distance^33^ is *f* = 0.25. To estimate conservative sample size, we used the smaller value (0.25). As we used a 2 (construal level: high vs. low) × 2 (relatedness of construal level manipulation: embedded vs. not embedded in AM retrieval) × 2 (narrative perspective: 1st-person vs. 3rd-person pronoun) mixed-factor design with all but narrative perspective as between-subject variables, we selected “ANOVA: repeated measures, within/between interaction measures” to run an a-priori power analysis in G*Power^34^. With effect size *f* = 0.25, *α*=0.05, number of groups = 4, number of repeated measurements = 2, power = 0.99, and correlation between repeated measures = 0.50, the analysis showed that a total sample size of more than 100 is required to detect an effect with power > 0.99. Thus, there should be more than 25 in each of the four groups.

### Participants

This study was approved by the Survey and Behavioural Research Ethics Committee at the Chinese University of Hong Kong (SBRE−19−283) and conducted in accordance with relevant guidelines and the Declaration of Helsinki. All participants provided written informed consent prior to their participation in the study.

We recruited 128 local Cantonese-speaking CUHK undergraduate students through advertisement in the university’s mass mail system. After removing participants with extreme scores on eudaimonic well-being, narrative word count (outside the range of ± 3 SD), or the memory age equaling zero or larger than 365 days as required by the instruction, we included data from 103 participants (52 females, mean age = 19.80, *SD* = 1.51) in the final analyses.

## Materials, design, and procedure

**Fig. 1:**
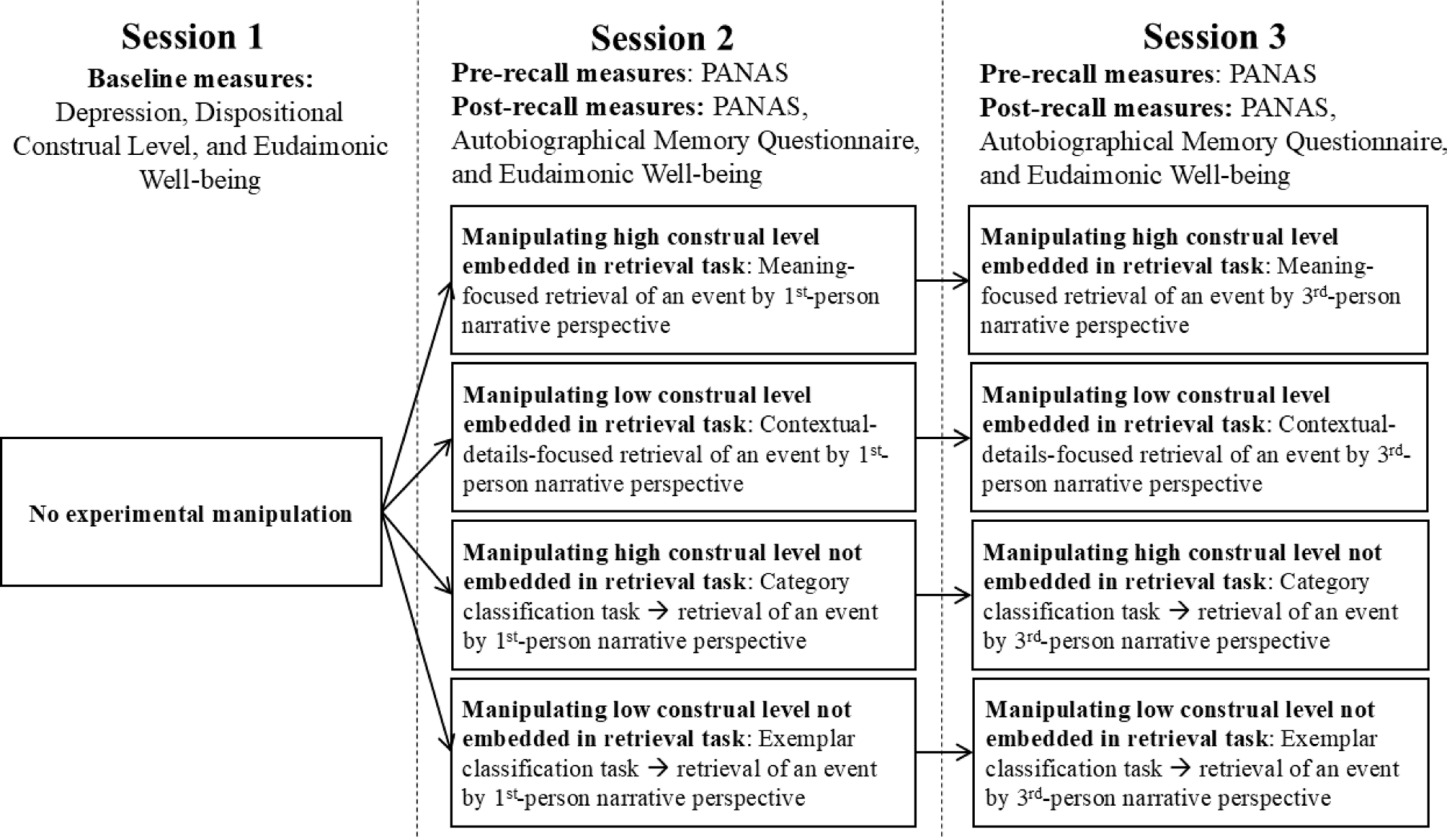
Procedure

The experiment adopts a 2 (construal level: high vs. low; between-subjects variable) × 2 (relatedness of construal level manipulation: embedded vs. not embedded in AM retrieval; between-subjects variable) × 2 (narrative perspective: 1st-person vs. 3rd-person pronoun; within-subjects variable) mixed-factor design. Figure 1 depicts the experimental procedures. Participants completed three sessions, with the average interval being 2.82 days (*SD* = 2.21) between Sessions 1 and 2 and 2.63 days (*SD* = 1.81) between Sessions 2 and 3.

**In Session 1**, we measured individual differences that might affect the post-recall affect and eudaimonic well-being *before* participants retrieved AM events and treated these as their baseline data. The scales developed in English were translated into Chinese using the back-translation method.

*Depression.* We considered self-reported depression symptoms because individual differences in depression related to the spontaneous usage of the 3rd-person over the 1st-person visual perspective^35^. Participants completed the 20-item Center for Epidemiologic Studies-Depression Scale (CES-D Scale)^36^. They provided ratings regarding the occurrence of feelings and behaviors over the previous week on a 4-point scale that ranged from 0 (rarely or none of the time: less than 1 day) to 3 (most or all of the time: 5–7 days). A higher score means more depressive experience. Cronbach’s *α* was 0.90.

*Dispositional construal level*. To measure and control for the individual differences in dispositional construal level (i.e., how participants construe behaviors in daily life), we used Behavior Identification Form^37^. Each item shows a behavior (e.g., reading) followed by two options, identifying that behavior at a low construal (how to do it, e.g., following lines of print) or a high construal (why doing it, e.g., gaining knowledge). Participants chose the one best describing the behavior. The choice of high-construal option scores “1” and that of low-construal option scores “0”. A greater cumulative score across 25 items indicates a higher dispositional construal level. Cronbach’s *α* was 0.72.

*Eudaimonic Well-being*. Following previous studies^38^, four scales were used to quantify multiple metrics of eudaimonic well-being. When we fit the data of the four scales using a second-order model or a bi-factor model, the data failed to converge, perhaps due to the insufficient sample size required for a structural equation model. Hence, we reported analyses on each variable separately.

The 9-item Chinese version of Rosenberg’s Self-Esteem Scale^39^ evaluates participants’ positive or negative views of themselves. Participants provided their ratings using a 4-point Likert scale, with responses ranging from 1 (strongly disagree) to 4 (strongly agree), to provide their ratings. Cronbach’s *α* was 0.92.

The 54-item Psychological Well-being Scale^40^ evaluates six dimensions: Self-acceptance, purpose in life, personal growth, autonomy, environmental mastery, and positive relations with others. Participants rated on a 7-point Likert scale from 1 = strongly disagree to 7 = strongly agree. Cronbach’s *α* was 0.92.

The 6-item Subjective Vitality Scale^41^ assesses the extent to which participants’ subjective feeling of aliveness and energy available to the self. Participants provided ratings on a 7-point Likert scale, with responses ranging from 1 (strongly disagree) to 7 (strongly agree). Cronbach’s *α* was 0.85.

The 10-item Meaning in Life Scale^42^ assesses individuals’ sense of meaning presence and their search for meaning in life. Participants used a 7-point Likert scale for their ratings, where 1 represented “strongly disagree” and 7 indicated “strongly agree”. Cronbach’s *α*s of the presence of meaning and search for meaning subscales were 0.87 and 0.83, respectively.

**In Session 2**, participants were randomly assigned to one of the four experimental conditions, as shown in Fig. 1. Participants, prior to AM retrieval, reported their current positive and negative affect on a 5-point Likert scale (1 = not at all; 5 = extremely) by completing the 10-item Positive Affect and Negative Affect Schedule (PANAS)^43^. Then, the event cuing technique was used to elicit the AM retrieval^44^, in which participants were instructed to recall an event that they experienced, that was important to them and brought them strong negative emotions at the time. To control for the temporal distances of the event that might cloud the effects of psychological distance on emotional intensity of the recalled event, we asked participants to recall the event that had occurred within the past year (12 months) and more than one day (24 h) ago. Please see Appendix A for the instructions to recall AM events. The construal level and psychological distance were manipulated as below.

**Manipulation of Construal Level.** As a manipulation of construal level *unrelated to* (or *not embedded in*) the AM retrieval task, participants were primed by doing a category or exemplar classification task prior to AM retrieval. Previous studies showed that the category or exemplar classification task is an effective manipulation to induce the priming effect of construal level on subsequent processing^47^. In the original task developed by Fujita et al.^45^, participants were presented with 40 everyday objects (e.g., soda, computer, pasta), each with two options. One option was an exemplar of the object, and the other option was the category to which the object belongs. We used a modified version of the task in which the options of the 40 objects were modified to make them more applicable to our current sample^48^. For example, in Fujita et al.’s version, the exemplar options of “newspaper” and “professor” were “the New York Times” and “Noam Chomsky”, respectively. In the new version, these were replaced with “Apple Daily” and “Joseph Sung”, respectively. Additionally, we augmented the stimulus pool by incorporating 40 additional objects, each with two options, ensuring participants received all 80 objects in the current study. Those in the high (or low) construal level priming condition were instructed to choose the option that refers to the object’s category (or an exemplar of the object). To ensure that participants were primed with the construal level as planned, those who did not reach 90% accuracy in this construal level priming task were replaced by additional participants.

As a manipulation of the construal level *related to* (or *embedded in*) the AM retrieval task, participants were probed to recall the contextual details or meaning of the events.

**Manipulation of Psychological Distance.** To vary psychological distance, we manipulated the narrative perspective when participants retrieved their AMs. In Session 2, participants used the 1st-person perspective to recall and narrate a negative AM event. At the end of AM retrieval, participants generated keywords for the event.

**Post-recall Measures.** After retrieving the event, participants completed the PANAS again to measure their current affect. Then they completed an Autobiographical Memory Questionnaire to rate the mnemonic characteristics of the recalled event, which was used in previous studies^49,50,51^. The characteristics include current emotional intensity (1 = not at all, 7 = extremely), emotional valence (1 = extremely negative, 7 = extremely positive), visual perspective (1 = my own eyes, 7 = an observer’s eyes), psychological distance (0 = I feel like the event happened today, 100 = I feel like the event occurred a very long time ago), memory importance (1 = not at all, 7 = extremely), imagery vividness (1 = not at all, 7 = extremely), retrieval easiness (1 = extremely difficult, 7 = extremely easy), memory age (temporal distance of the memory in days), and transitional impacts in material and psychological states (1 = not at all, 7 = extremely). As we did not propose specific hypotheses regarding all the mnemonic characteristics of the recalled event except emotional intensity, we only reported the results on the emotional intensity of the recalled event in the main text and provided the results on other characteristics in Appendix B. In addition, participants completed the same eudaimonic well-being measures as those in Session 1. The order of the eudaimonic well-being measures and Autobiographical Memory Questionnaire was counterbalanced between participants.

**In Session 3**, the procedure and measures were the same as in Session 2, with the exception that participants were required to recall the same event using the 3rd-person perspective instead of the 1st-person perspective. In the retrieval task, participants were presented with the keywords they wrote down in Session 2 as a reminder of the event. At the end of the experiment, participants were debriefed and paid HK$300 for compensation.

## Results

Unless explicitly stated otherwise, the significant level was set at *p* < .05. Table 1 displays the means and standard deviations of all dependent variables in each condition.

**Table 1:** Means (standard deviations) of AM contents, AM characteristics, eudaimonic well-being, and affect.

		Low construal level manipulation not embedded in retrieval task (*N* = 27)	Low construal level manipulation embedded in retrieval task (*N* = 25)	High construal level manipulation not embedded in retrieval task (*N* = 25)	High construal level manipulation embedded in retrieval task (*N* = 26)
**AM contents**					
Background information	Session 2: 1st-person perspective	5.65 (3.58)	4.66 (3.27)	4.50 (3.66)	6.63 (3.65)
	Session 3: 3rd-person perspective	4.63 (2.94)	4.32 (3.23)	4.24 (3.71)	5.08 (3.18)
Psychological states	Session 2: 1st-person perspective	7.30 (4.09)	9.30 (4.33)	8.78 (3.00)	9.52 (5.44)
	Session 3: 3rd-person perspective	6.93 (5.33)	7.70 (4.66)	6.82 (4.30)	8.33 (5.45)
Physical actions	Session 2: 1st-person perspective	8.15 (5.70)	7.46 (4.64)	4.38 (4.34)	5.13 (3.67)
	Session 3: 3rd-person perspective	7.63 (4.40)	6.86 (4.14)	4.94 (4.28)	3.75 (2.43)
Perceptual information	Session 2: 1st-person perspective	6.07 (4.54)	6.18 (5.60)	6.44 (5.63)	5.02 (5.88)
	Session 3: 3rd-person perspective	4.63 (3.38)	5.00 (4.00)	4.42 (3.41)	3.04 (3.12)
Personal meaning and significance	Session 2: 1st-person perspective	1.61 (1.37)	1.94 (1.83)	1.38 (1.04)	9.71 (5.31)
	Session 3: 3rd-person perspective	1.44 (1.26)	1.24 (1.29)	1.26 (1.31)	8.38 (5.22)
1st-person pronouns	Session 2: 1st-person perspective	12.71 (7.45)	16.10 (11.10)	11.20 (5.47)	15.98 (8.39)
	Session 3: 3rd-person perspective	0.39 (0.94)	0.04 (0.20)	0.04 (0.20)	0.77 (2.30)
3rd-person pronouns	Session 2: 1st-person perspective	4.02 (5.02)	5.66 (6.81)	3.34 (3.60)	4.85 (5.41)
	Session 3: 3rd-person perspective	12.80 (7.16)	14.94 (8.23)	12.24 (5.18)	15.94 (10.74)
**Self-reported measures**					
Emotional intensity of AM event	Session 2: 1st-person perspective	4.67 (1.27)	4.88 (1.45)	4.92 (1.12)	4.69 (1.19)
	Session 3: 3rd-person perspective	4.00 (1.49)	3.84 (1.40)	3.72 (1.72)	3.81 (1.27)
Self-esteem	Session 2: 1st-person perspective	23.89 (4.69)	23.24 (7.62)	24.16 (6.17)	25.19 (5.58)
	Session 3: 3rd-person perspective	23.70 (4.62)	24.68 (7.08)	24.96 (5.83)	25.35 (4.99)
Psychological well-being	Session 2: 1st-person perspective	238.33 (26.67)	233.40 (41.38)	242.12 (43.57)	246.58 (29.80)
	Session 3: 3rd-person perspective	242.74 (26.54)	241.44 (41.67)	245.16 (45.03)	246.08 (23.56)
Subjective vitality	Session 2: 1st-person perspective	21.30 (5.78)	22.40 (6.83)	23.36 (6.45)	26.19 (5.31)
	Session 3: 3rd-person perspective	21.44 (6.85)	24.52 (7.42)	23.80 (7.17)	25.31 (6.19)
Presence of meaning	Session 2: 1st-person perspective	19.52 (6.35)	19.88 (6.67)	20.20 (7.66)	20.96 (5.17)
	Session 3: 3rd-person perspective	19.56 (6.42)	20.48 (6.10)	19.88 (7.06)	22.27 (5.44)
Searching for meaning	Session 2: 1st-person perspective	25.63 (4.40)	25.92 (3.80)	28.08 (4.52)	27.65 (3.27)
	Session 3: 3rd-person perspective	26.19 (4.87)	26.00 (4.18)	27.64 (4.64)	27.38 (4.01)
Pre-recall positive affect	Session 2: 1st-person perspective	25.22 (8.32)	24.04 (7.63)	26.36 (7.05)	25.65 (6.61)
	Session 3: 3rd-person perspective	23.00 (7.66)	22.12 (8.31)	21.48 (7.18)	22.04 (7.35)
Pre-recall negative affect	Session 2: 1st-person perspective	22.41 (6.49)	17.88 (6.06)	22.92 (9.64)	17.69 (5.40)
	Session 3: 3rd-person perspective	20.33 (7.66)	15.92 (6.20)	17.72 (9.48)	14.96 (5.53)
Post-recall positive affect	Session 2: 1st-person perspective	22.78 (6.74)	21.12 (6.00)	21.68 (6.48)	23.50 (7.60)
	Session 3: 3rd-person perspective	20.70 (6.75)	22.04 (7.97)	21.04 (8.21)	21.19 (7.67)
Post-recall negative affect	Session 2: 1st-person perspective	22.26 (7.61)	22.40 (8.74)	22.68 (8.59)	17.00 (4.53)
	Session 3: 3rd-person perspective	20.78 (7.47)	18.76 (6.92)	19.48 (9.21)	14.77 (5.62)
**Baseline measures in Session 1**					
Depression	No manipulation of psychological distance	41.30 (9.81)	39.56 (12.20)	37.36 (9.35)	36.85 (7.84)
Dispositional construal level		15.15 (4.03)	13.96 (3.96)	13.76 (3.73)	14.54 (4.40)
Self-esteem		23.96 (4.82)	24.00 (6.57)	25.28 (6.34)	25.62 (4.97)
Psychological well-being		240.00 (23.70)	236.20 (35.28)	248.52 (43.08)	243.92 (23.27)
Subjective vitality		23.48 (5.60)	24.24 (6.30)	25.96 (6.77)	26.42 (6.37)
Presence of meaning		19.56 (7.04)	20.00 (6.57)	20.48 (6.69)	21.77 (5.84)
Searching for meaning		25.96 (4.28)	27.92 (4.25)	27.76 (4.82)	27.58 (3.72)

### Content analysis and manipulation check

All memories written in Sessions 2 and 3 were coded to verify whether they aligned with the experimental manipulations. The AM descriptions at the low construal level are supposed to contain more content about details than those at the high construal level, whereas the AM descriptions at the high construal level, particularly those focused on event meaning, presumably contain more expressions about personal meaning and significance drawn from the event. The 1st-person (or 3rd-person) perspective is expected to elicit more frequent use of 1st-person (or 3rd-person) personal pronouns.

We arranged all memories written in Sessions 2 and 3 in a randomized order and distributed them to two raters, who worked independently and did not know the hypotheses or experimental conditions of the narrations. They classified each sentence separated by commas/periods in participants’ narrations into one of the five categories, which were adopted and modified based on previous work^52^. (a) Background information: The background of an event, typically appearing at the beginning of a narration, e.g., last summer, when I was in junior high school. (b) Psychological states: Descriptions or inferences of one’s own or others’ mental states, or expressions of opinions, attitudes, and speculations, e.g., I doubted my abilities, I was curious about why they did that. (c) Physical actions: Actions or behaviors performed by oneself or others, e.g., I passed the ball to him; she exerted force to move the lever. (d) Perceptual information: Directly visual, auditory, or sensory contents to describe the surrounding environment; or one’s own or others’ speech or dialogue, e.g., sometimes there was a lake or a river; I saw my name on the report. (e) Personal meaning and significance: Expressions about the significance and impact of the event on oneself or others, e.g., I still can’t forget this event now; it was a significant moment in my life. The raters also coded words in the narrations for 1st-person accounts (e.g., I, me, my, mine) or 3rd-person accounts (e.g., he/she, his/him/her). Overall, the raters summed the number and assigned a score for each of the above seven categories. The Intraclass Correlation Coefficients (ICCs) between the two coders for each category ranged from 0.91 to 1.00.

Given the high inter-rater reliability, we used the mean score of each coded category, averaged across two raters, as the dependent variable in a separate statistical model. We fitted 2 (construal level) × 2 (narrative perspective) × 2 (relatedness of construal level manipulation) repeated-measures general linear models (GLMs). Table 2 shows the statistics of these analyses. Results showed that the narrations at the low construal level had more descriptions of physical actions and contextual details than those at the high construal level, regardless of whether the construal level manipulation is related to AM retrieval or not. Narrations from the 1st-person perspective involved more background information, perceptual information, psychological states, personal meaning and significance, and 1st-person pronouns, but fewer 3rd-person pronouns compared to those from the 3rd-person perspective. Moreover, we found that the interaction between construal level and manipulation relatedness was significant in the category of personal meaning and significance. Simple-effect analyses showed that when construal level manipulation was embedded in AM retrieval, narrations contained more personal meaning and significance in the high (vs. low) construal level condition, *F*(1,99) = 101.06, *p* < .001. In contrast, when the construal level was primed by category/exemplar classification task, i.e., not embedded in AM retrieval, narrations at the high and low construal levels did not differ in personal meaning and significance, *F*(1,99) = 0.08, *p* = .78, suggesting that narrations had more personal meaning and significance in high construal level condition *only* when the construal level manipulation was embedded in AM retrieval. In summary, the above findings support the effective manipulations of narrative perspective, construal level, and manipulation relatedness.

**Table 2:** The effects of construal level, manipulation relatedness, narrative perspective, and their interactions on AM contents, emotional intensity of AM event, eudaimonic well-being, and post-recall affect.

	^Construal level^		^Manipulation relatedness^		^Narrative perspective^		^CL × MR^		^CL × NP^		^NP × MR^		^CL × MR × NP^	
	^*F*^	^*MS*^	^*F*^	^*MS*^	^*F*^	^*MS*^	^*F*^	^*MS*^	^*F*^	^*MS*^	^*F*^	^*MS*^	^*F*^	^*MS*^
^**AM Contents**^														
^Background information^	^0.23^	^4.58^	^0.46^	^9.01^	^8.94**^	^**32.44**^	^2.99^	^58.61^	^0.19^	^0.68^	^0.34^	^1.23^	^3.46^	^12.56^
^Psychological states^	^0.45^	^15.9^	^2.27^	^81.16^	^11.16**^	^**84.38**^	^0.03^	^0.91^	^0.59^	^4.49^	^0.09^	^0.69^	^1.7^	^12.83^
^Physical actions^	^14.55***^	^**454.82**^	^0.37^	^11.52^	^2.17^	^12.14^	^0.11^	^3.36^	^0.05^	^0.28^	^2.36^	^13.2^	^1.99^	^11.16^
^Perceptual information^	^0.81^	^28.29^	^0.5^	^17.4^	^21.23***^	^**141.14**^	^0.99^	^34.56^	^0.92^	^6.1^	^0.05^	^0.3^	^0.03^	^0.16^
^Personal meaning and significance^	^48.19***^	^**676.11**^	^55.64***^	^**780.57**^	^6.18*^	^**17.21**^	^53.87***^	^**755.83**^	^0.39^	^1.08^	^3.5^	^9.74^	^0.52^	^1.46^
^1st-person pronouns^	^0.14^	^4.97^	^6.62*^	^**235.47**^	^271.91***^	^**9637.27**^	^0.55^	^19.5^	^0.36^	^12.9^	^5.52*^	^**195.46**^	^0.01^	^0.3^
^3rd-person pronouns^	^0.05^	^3.52^	^3.62^	^260.09^	^210.19***^	^**4656.32**^	^0.09^	^6.51^	^0.55^	^12.08^	^1.06^	^23.41^	^0.42^	^9.23^
^**Self-reported measures**^														
^Emotional intensity of AM event^	^0.58^	^1.43^	^0.33^	^0.8^	^**3.80†**^	^**4.24**^	^0.03^	^0.07^	^0.4^	^0.44^	^0.23^	^0.25^	^1.45^	^1.61^
^Self-esteem^	^0.21^	^10.33^	^0.51^	^24.74^	^0.55^	^2.44^	^0.02^	^0.81^	^0.01^	^0.02^	^0.58^	^2.58^	^3.52^	^15.68^
^Psychological well-being^	^0.22^	^366.38^	^2.64^	^4320.32^	^0.04^	^5.03^	^0.1^	^169.85^	^4.41*^	^**605.33**^	^0.02^	^3.28^	^0.62^	^84.65^
^Subjective vitality^	^4.14*^	^**199.29**^	^0.41^	^19.46^	^1.43^	^14.05^	^0.07^	^3.31^	^3.06^	^30.1^	^0.02^	^0.23^	^3.24^	^31.87^
^Presence of meaning^	^0.07^	^3.89^	^0.01^	^0.36^	^1.14^	^6.09^	^0.11^	^6.18^	^0.06^	^0.31^	^3.56^	^19.05^	^1.28^	^6.87^
^Searching for meaning^	^5.68*^	^**168.89**^	^0.04^	^1.04^	^4.86*^	^**23.24**^	^0.2^	^6.05^	^2.36^	^11.28^	^0.2^	^0.94^	^0.4^	^1.92^
^Post-recall positive affect^	^0.49^	^10.79^	^0.75^	^16.51^	^0.3^	^3.07^	^0.004^	^0.09^	^0.16^	^1.65^	^0.01^	^0.08^	^7.23**^	^**75.24**^
^Post-recall negative affect^	^5.45*^	^**157.83**^	^0.23^	^6.57^	^1.42^	^26.71^	^10.85**^	^**313.94**^	^0.18^	^3.3^	^<0.001^	^0.01^	^1.06^	^20.08^

Note: * *p*<.05, ** *p*<.01, *** *p*<.001, † *p*=.05; *df*=(1, 95); MS = Mean Square, NP = Narrative Perspective, CL = Construal Level, MR = Manipulation Relatedness. Across Sessions 2 and 3, the narrative perspective was manipulated as a within-subject variable.

### Baseline data at Session 1

We fitted a 2 (construal level) × 2 (relatedness of construal level manipulation) GLM with the baseline variables at the first session as dependent variables. Neither the main effects nor the interactions were significant (see Table 3), suggesting no difference among the four experimental groups in depression, dispositional construal level, self-esteem, psychological well-being, subjective vitality, or meaning in life in the baseline data. Therefore, participants were effectively randomized when assigned to one of the four groups, and homogeneous values were shown at the baseline measures across the experimental groups.

**Table 3:** The main effects of construal level, manipulation-relatedness, and their interactions on baseline measures (i.e., depression, dispositional construal level, and eudaimonic well-being) in Session 1.

	Construal level		Manipulation relatedness		CL × MR	
	*F*	*MS*	*F*	*MS*	*F*	*MS*
Depression	2.90	284.40	0.33	32.56	0.10	9.61
Dispositional construal level	0.26	4.22	0.07	1.08	1.52	24.87
Self-esteem	1.70	55.30	0.03	0.89	0.02	0.57
Psychological well-being	1.64	1696.72	0.44	453.43	0.004	4.08
Subjective vitality	3.57	139.75	0.24	9.60	0.01	0.56
Presence of meaning	1.09	46.66	0.45	19.33	0.11	4.59
Searching for meaning	0.74	13.59	1.10	20.24	1.61	29.45

Note: MS = Mean Square, CL = Construal Level, MR = Manipulation Relatedness.

#### Impacts of construal level and manipulation relatedness on emotional intensity of AM event, eudaimonic well-being, and affect in Session 2

To test the hypotheses about the effects of construal level (Hypotheses a and d), we focused on the data of Session 2 when participants recalled AM events from the 1st-person perspective and fitted 2 (construal level) × 2 (relatedness of construal level manipulation) GLMs. The emotional intensity of AM events, eudaimonic well-being measures, or post-recall positive and negative affect were entered as dependent variables in separate models. In all models, the pre-recall positive and negative affect in Session 2 were entered as covariates. Table 4 shows the statistics of these analyses.

First, the main effect of construal level was not significant on emotional intensity. Second, the main effect of construal level was significant on subjective vitality and search for meaning: participants reported higher levels of subjective vitality and search for meaning, reflecting eudaimonic well-being, in the high (vs. low) construal level condition. However, no significant effect of construal level was found regarding self-esteem, psychological well-being, or the presence of meaning. Therefore, Hypothesis a was only partially supported.

**Table 4:** The main effects of construal level, manipulation-relatedness, and their interactions on emotional intensity of the recalled event, eudaimonic well-being, and post-recall affect in Session 2.

	Construal level		Manipulation relatedness		CL × MR	
	*F*	*MS*	*F*	*MS*	*F*	*MS*
Emotional intensity of AM event	0.02	0.02	0.01	0.02	0.91	1.38
Self-esteem	0.31	8.58	0.74	20.71	0.36	10.16
Psychological well-being	0.83	789.19	1.93	1831.22	0.30	286.39
Subjective vitality	5.14*	**150.25**	0.58	16.93	0.39	11.29
Presence of meaning	0.07	2.14	0.13	4.07	<0.001	0.004
Searching for meaning	6.27*	**102.59**	<0.001	0.01	0.22	3.58
Post-recall positive affect	0.10	2.10	0.67	13.67	3.15	64.35
Post-recall negative affect	5.80*	**200.79**	0.06	1.90	5.57*	**192.83**

Note: * *p*<.05; MS=Mean Square, CL=Construal Level, MR=Manipulation Relatedness; df=(1,97); In Session 2, the narrative perspective was manipulated by the 1st-person perspective in all groups.

Second, the main effect of construal level on post-recall negative affect was significant and qualified by a significant construal level × manipulation relatedness interaction. Simple-effect analyses showed that when the construal level manipulation was embedded in AM retrieval, participants reported weaker negative affect at a high (vs. low) construal level, *F*(1,97) = 11.25, *p* < .01. However, when construal level manipulation was not embedded in AM retrieval, participants reported similar negative affect at high and low construal levels, *F*(1,97) = 0.02, *p* = .97. When construal level manipulation was related to AM retrieval, participants who focused on the meaning rather than contextual details of the AM events reported weaker negative affect, demonstrating the mitigating effect of high construal level on participants’ negative affect after AM retrieval, consistent with Hypothesis d.

#### Impacts of construal level, manipulation relatedness, and narrative perspective on emotional intensity of AM event, eudaimonic well-being, and affect

We analyzed the data from Sessions 2 and 3 to test the effects of shifting narrative perspective from 1st-person to 3rd-person and its associated interactions; i.e., our Hypotheses b, c, and d. We fitted 2 (construal level) × 2 (relatedness of construal level manipulation) × 2 (narrative perspective) repeated-measures GLMs. The emotional intensity of AM event, each measure of eudaimonic well-being, and post-recall positive or negative affect was entered as the dependent variable in separate models. Pre-recall positive or negative affect in Sessions 2 and 3 were entered as covariates in all models. Table 2 presents the statistics of the analyses.

For the perceived emotional intensity of AM events, the effect of narrative perspective was marginally significant, *p* = .054, based on the *p*-value of 0.05 criterion. As the directional test (one-tailed test) should be used to evaluate the effect reported in the literature^14^, we consider it significant. For eudaimonic well-being measures, we found significant main effects of narrative perspective and construal level on meaning search and a significant main effect of construal level on subjective vitality. Thus, in line with Hypotheses a and b, narrating negative AM events in the 3rd-person perspective reduced the emotional intensity of the event and increased the search for life meaning compared to doing that in the 1st-person perspective, and narrating AM events at a high construal level led to higher levels of subjective vitality and search for life meaning than doing that at a low construal level.

We also found a significant construal level × psychological distance interaction on psychological well-being. While simple-effect analyses did not show significant effects of construal level in the 1st- or 3rd-person perspective condition, the effect of high construal level on boosting psychological well-being was *larger* in the 1st-person perspective condition [6.27, *F*(1,95) = 0.99, *p* = .32] than in the 3rd-person perspective condition [−0.78, *F*(1,95) = 0.02, *p* = .89]. While the interaction supported the conceptual link between psychological distance and construal level, the effect of construal level on psychological well-being was stronger, but not weaker, for events with closer psychological distance (i.e., events narrated in the 1st-person perspective), contrary to the prediction of the Construal Level Theory.

For post-recall positive affect, we found a significant three-way interaction among construal level, relatedness of construal level manipulation, and narrative perspective. We then ran 2 (construal level) × 2 (narrative perspective) repeated-measures GLMs with pre-recall positive and negative affect as covariates, separately by manipulation-relatedness. Results showed that the construal level × narrative perspective interaction was significant when the construal level manipulation was embedded in AM retrieval, *F*(1,45) = 4.22, *MS* = 49.19, *p* = .046, but not when it was not. Simple-effect analyses showed that the effect of high construal level on increasing post-recall positive affect was larger for events narrated in the 1st-person perspective [1.57, *F*(1,45) = 1.69, *p* = .20] than those narrated in the 3rd-person perspective [−1.25, *F*(1,45) = 0.93, *p* = .34]. The findings were again opposite to the prediction of Construal Level Theory that the effect of high construal level on increasing positive affect might be stronger for events narrated in the 3rd-person perspective.

For post-recall negative affect, the significant effect of construal level was qualified by a significant interaction between construal level and manipulation relatedness. Simple-effect analyses showed that when the construal level manipulation was embedded in AM retrieval, the high construal level led to weaker negative affect than the low construal level *F*(1,99) = 6.22, *p* < .05. However, when the construal level manipulation was not embedded in AM retrieval, there was no difference in the effect of construal level on post-recall negative affect, *F*(1,99) = 0.06, *p* = .82. Thus, as predicted by Hypothesis d, the effect of construal level was only significant when the construal level manipulation was embedded in AM retrieval.

## Discussion

The current study aimed to investigate the effects of psychological distance, construal level, and their interactions on the perceived emotional intensity of negative AM events and participants’ post-recall affect and eudaimonic well-being. Regarding the effects of narrative perspective, we found that shifting narrative perspective from 1st-person to 3rd-person during AM retrieval led to reduced emotional intensity, which replicated the effects of narrative perspective on AM characteristics reported by Gu and Tse^14^. We extended the previous finding in two ways. First, the effects of shifting narrative perspective on the emotional intensity of AM event were robust whether high or low construal level was primed before the AM retrieval task or induced via instructions during AM retrieval. Second, the change of narrative perspective at AM retrieval from 1st-person to 3rd-person contributed to the increase in search for life meaning after retrieval, i.e., far psychological distance led to more search for meaning than close psychological distance.

The results uncovered both main effects of construal level and its interaction with psychological distance, which holds strong theoretical implications for Construal Level Theory. First, retrieving the AM events at a high construal level—focus on the event meaning—yielded greater eudaimonic well-being, as indicated by higher subjective vitality, greater search for meaning in life, and reduced post-recall negative affect, compared to retrieving the events at a low construal level (i.e., focus on contextual details). These extend the influence of construal level beyond cognitive processes into the domain of emotion. Second, we obtained a significant interaction between construal level and psychological distance on psychological well-being, but inconsistent with the Construal Level Theory, the effect of high construal level on boosting psychological well-being was *stronger* when events were narrated in the 1st-person perspective than in the 3rd-person perspective. This interaction pattern challenges the assumption of a parallel correspondence between construct level and psychological distance, suggesting a boundary condition for the conceptual link between the two constructs^53^. In addition, we examined the relatedness of construal level manipulation to AM retrieval. Results indicated that high construal level reduced post-recall negative affect only when the construal level manipulation was embedded within the AM retrieval task, rather than when it was primed by the category/exemplar classification task. These findings highlight that not all manipulations of construal level are equally effective—at least in the context of AM retrieval—underscoring the importance of task-related relevance in construal level interventions.

Our findings further demonstrate that the construal level and narrative perspective adopted during AM retrieval can significantly influence both the emotional intensity of the recalled events and individuals’ eudaimonic well-being. These results have important implications for the theoretical understanding and practical application of expressive writing interventions^54,55^. Previous research has highlighted that psychological distancing can attenuate negative affect and enhance well-being when individuals retrieve adverse personal experiences, in which psychological distancing took various forms, such as self-distanced expressive writing about interpersonal experiences^56^, adopting an observer perspective in mind on the self^57^, or employing psychologically distant language^14,58,59^. The current study extends these paradigms by showing that shifting the narrative perspective from 1st-person to 3rd-person during retrieval of negative AM events can lower emotional intensity and increase the search for meaning in life. At the same time, our findings reveal that psychological benefits are not limited to the adoption of a psychologically distant perspective. Specifically, individuals may also experience enhanced psychological well-being when using the 1st-person perspective, provided that they emphasize high-construal, meaning-based information in their narrations. This suggests that both self-distancing and self-immersion can serve therapeutic functions, depending on the construal orientation being adopted. Taken together, our findings provide a conceptual basis for developing high-construal, meaning-based AM interventions aimed at promoting health and eudaimonic well-being, particularly among young adults. Future research should seek to replicate these effects and also examine their generalizability to other populations, such as older adults or individuals experiencing depressive symptoms.

Before concluding, we discussed the potential limitations of our experiment and suggested future directions. One issue related to the narrative perspective manipulation is the fixed order in the narrative perspective that was only shifted from 1st- to 3rd-person. It could be argued that the decrease in emotional intensity (or increase in search for meaning) was due to the repeated retrieval of the same AM events, rather than the switch from the 1st-person to 3rd-person perspective. Previous research reported that the decrease in emotional intensity did not occur when the narrative perspective was shifted from 3rd- to 1st- person^14,31,60^. However, it is an empirical question whether retrieving a negative AM event repeatedly (with the same or a switched perspective) could enhance or undermine eudaimonic well-being.

One could argue that the effects of construal level and narrative perspectives on eudaimonic well-being or post-recall affect were solely due to the quality of retrieved AM, e.g., participants might find it easier to recall the meaning of AM events compared to their contextual details, resulting in lower post-recall negative affect in the high (vs. low) construal level condition. However, there was no difference in AM characteristics, including retrieval easiness, memory importance, or memory age across all conditions (see Appendix B), suggesting that these characteristics might not significantly compromise the interpretation of our findings.

Notably, the effect of construal level, narrative perspective, or the interaction was not consistently significant on all measures of eudaimonic well-being or affect. These measures could not be combined into a single factor, suggesting that they were different in a certain way and reflected different aspects of eudaimonic well-being and affect. Despite not being significant, most of the effects of construal level on other measures of eudaimonic well-being and affect were in the predicted direction (see Table 1), except for post-recall positive affect in the 3rd-person perspective condition. This direction was especially evident when the construal level instruction was embedded in AM retrieval. Similarly, the effects of narrative perspective on those measures were also generally in the predicted direction. Future research should identify the specific aspects of eudaimonic well-being that are sensitive to the effects of construal level and narrative perspective. Besides, the interplay between construal level and psychological distance did not consistently demonstrate an effect across all measures of eudaimonic well-being, providing limited support for the intertwined relationship between construal level and psychological distance posited by the Construal Level Theory.

The current research focused on eudaimonic well-being, which is associated with viewing one’s life with meaning, purpose, and a sense of growth. Future studies could investigate other conceptualizations and measures of well-being. For example, hedonic well-being could be measured via Diener et al.’s Satisfaction With Life Scale^61^. It is possible that hedonic well-being would be more sensitive to the influence of lower construal level, in contrast to the eudaimonic well-being in the current study. In addition, more comprehensive well-being scales could be employed, such as the PERMA-Profiler scale^62^,which measures the multidimensionality of well-being, including positive emotions, engagement, relationships, meaning, and accomplishment. By using alternative conceptualizations and measures of well-being, researchers can test whether the current findings generalize to multi-constructs of well-being.

Finally, the current manipulation of narrative perspective is not a conventional method for manipulating psychological distance. However, we did establish a relationship between narrative perspective and psychological distance as perceived by the participants. Future studies should consider manipulating other dimensions of psychological distance during AM retrieval, such as by comparing events from proximal vs. distal spatial distance (e.g., events that occurred in one’s home region vs. distance location) or from close or far social distance (e.g., events that happened to oneself vs. events that happened to friends). Importantly, while these manipulations of spatial or social distances may involve recalling different events, our current approach uniquely manipulated perceived distance within the same memory, offering a more controlled method for examining its effects.

## Conclusion

The current study represents novel findings, revealing the distinct and interactive effects of construal level and psychological distance during AM retrieval on the emotional intensity of AM event, as well as participants’ post-recall affect and eudaimonic well-being. By orthogonally manipulating construal level and narrative perspective, we demonstrated that retrieving negative AMs at a high construal level led to higher subjective vitality, more search for meaning in life, and lower post-recall negative affect than a low construal level. We also found that distant psychological distance (as manipulated by the 3rd-person perspective) led to reduced emotional intensity of negative AM events and more search for life meaning than close psychological distance (as manipulated by the 1st-person perspective). Furthermore, we found that the effects of construal level were moderated by narrative perspective: the benefit of high construal level on boosting psychological well-being was more pronounced when participants used the 1st-person perspective rather than the 3rd-person perspective, challenging the assumption of a parallel relationship between high construal and psychological distance held by Construal Level Theory. This study advances our understanding of how cognitive framing during memory retrieval can regulate emotion and support eudaimonic well-being. These findings offer theoretical insights into Construal Level Theory and practical implications for high-construal, meaning-based interventions to support emotional regulation and well-being.

## Electronic supplementary material

Below is the link to the electronic supplementary material.

Supplementary Material 1

Supplementary Material 2

## Data Availability

Data reported in the paper are available at: https://osf.io/hgzru/files/osfstorage?view_only=c3400b2c7ff447fa8f2b0163e9c2f1d9.

## Footnotes

1 We acknowledged one reviewer for pointing out an alternative explanation for this result: Evaluating strangers may involve different processes with constructing one’s own values (e.g., liking or sympathy).
