# Supplementary material for: Shifting narrative perspective and construal level shape emotional response and enhance eudaimonic well-being
Source: Sci Rep. 2025 Jun 5;15:19778. doi: 10.1038/s41598-025-01946-8 (PMC12141604; doi:10.1038/s41598-025-01946-8)
Supplement: Supplementary file 1 — Supplementary Material 1 [file 41598_2025_1946_MOESM1_ESM.docx]

**Appendix A: The instructions to recall autobiographical memory in the 1^st^-person narrative perspective condition.**

The instructions were the same in the 3^rd^-person perspective condition except that the participants wrote down the event in the 3^rd^-person pronoun he or she. The traditional Chinese version was used in the current study, based on which the English version was translated.

1. **For participants in the *high* construal level *related* to autobiographical memory retrieval.**

對於發生在自己身上的一件往事，我們既可以採用第一人稱“我”的方式來回憶它，也可以採用第三人稱“他/她”的方式來回憶。比如，當我們回憶某天早晨去公園的經歷時，我們可以說，“我穿過馬路，來到了附近的公園。我看到了廣場上有一群做晨操的人，我走了過去……”，也可以說成“他/她穿過馬路，來到了附近的公園。他/她看到了廣場上有一群做晨操的人，他/她走了過去……”

接下來，請你按照指示完成回憶任務和回答相應的問題。

**回憶任務**

請回憶一件你親身經歷過的，對你來說重要的事件，這件事給當時的你帶來了強烈的負面情緒。這件事發生在過去的一年（12個月）之內，1天（24小時）之前。

**請詳細説明事件對你的意義、價值和含義，例如你從事件中學到了什麽、事件對你的影響，以及爲什麽這是你一生中重要的事件。**

請用第一人稱“我”寫下這件事。你有10分鐘的時間回憶並記錄下這件事。

For a specific memory that once happened to us, we can recall it in a 1^st^ -personal pronoun “I” or in a 3^rd^-personal pronoun “he/she”. For example, when remembering a trip to a park in the morning, we can recall the event in a 1^st^-personal pronoun and write “I crossed the street and came to a park. I saw a group of people doing morning exercise on the square, and I walked over...” or we can also recall the same event in a 3^rd^-personal pronoun and write “He/She crossed the street and came to a park. He/She saw a group of people doing morning exercise on the square, and he/she walked over...”

Next, please follow the instructions below to complete the retrieval task and answer the corresponding questions:

**Retrieval Task**

Please recall an event that you personally experienced, one that was important to you and brought you strong negative emotions at the time. This event must have occurred within the past year (12 months) but more than one day (24 hours) ago.

**Please explain in detail the meaning, value, and significance of this event. For instance, what have you learned from it, how has it affected you, and why is it important in your life?**

Please use the 1^st^-person pronoun “I” to write down this event. You have 10 minutes to recall and write down this event.

1. **For participants in the *low* construal level *related* to autobiographical memory retrieval.**

對於發生在自己身上的一件往事，我們既可以採用第一人稱“我”的方式來回憶它，也可以採用第三人稱“他/她”的方式來回憶。比如，當我們回憶某天早晨去公園的經歷時，我們可以說，“我穿過馬路，來到了附近的公園。我看到了廣場上有一群做晨操的人，我走了過去……”，也可以說成“他/她穿過馬路，來到了附近的公園。他/她看到了廣場上有一群做晨操的人，他/她走了過去……”

接下來，請你按照指示完成回憶任務和回答相應的問題。

請回憶一件你親身經歷過的，對你來說重要的事件，這件事給當時的你帶來了強烈的負面情緒。這件事發生在過去的一年（12個月）之內，1天（24小時）之前。

**請詳細説明事件的背景資料，例如事件發生的地點、時間，參與事件的人和物，以及你和其他人的行爲、反應及其角色，並且説明你的反應和/或行為的結果。**

請用第一人稱“我”寫下這件事。你有10分鐘的時間回憶並記錄下這件事。

For a specific memory that once happened to us, we can recall it in a 1^st^ -personal pronoun “I” or in a 3^rd^-personal pronoun “he/she”. For example, when remembering a trip to a park in the morning, we can recall the event in a 1^st^-personal pronoun and write “I crossed the street and came to a park. I saw a group of people doing morning exercise on the square, and I walked over...” or we can also recall the same event in a 3^rd^-personal pronoun and write “He/She crossed the street and came to a park. He/She saw a group of people doing morning exercise on the square, and he/she walked over...”

Next, please follow the instructions below to complete the retrieval task and answer the corresponding questions:

**Retrieval Task**

Please recall an event that you personally experienced, one that was important to you and brought you strong negative emotions at the time. This event must have occurred within the past year (12 months) but more than one day (24 hours) ago.

**Please explain in detail the background information of this event. For instance, the location and time of the event; the people and objects involved in the event; the behaviors, reactions, and roles of you and others; and the outcome of your reaction or behavior.**

Please use the 1^st^-person pronoun “I” to write down this event. You have 10 minutes to recall and write down this event.

1. **For participants in the *high* and *low* construal levels *unrelated* to autobiographical memory retrieval.**

對於發生在自己身上的一件往事，我們既可以採用第一人稱“我”的方式來回憶它，也可以採用第三人稱“他/她”的方式來回憶。比如，當我們回憶某天早晨去公園的經歷時，我們可以說，“我穿過馬路，來到了附近的公園。我看到了廣場上有一群做晨操的人，我走了過去……”，也可以說成“他/她穿過馬路，來到了附近的公園。他/她看到了廣場上有一群做晨操的人，他/她走了過去……”

接下來，請你按照指示完成回憶任務和回答相應的問題。

**回憶任務**

請回憶一件你親身經歷過的，對你來說重要的事件，這件事給當時的你帶來了強烈的負面情緒。這件事發生在過去的一年（12個月）之內，1天（24小時）之前。

請用第一人稱“我”寫下這件事。你有10分鐘的時間回憶並記錄下這件事。

For a specific memory that once happened to us, we can recall it in a 1^st^ -personal pronoun “I” or in a 3^rd^-personal pronoun “he/she”. For example, when remembering a trip to a park in the morning, we can recall the event in a 1^st^-personal pronoun and write “I crossed the street and came to a park. I saw a group of people doing morning exercise on the square, and I walked over...” or we can also recall the same event in a 3^rd^-personal pronoun and write “He/She crossed the street and came to a park. He/She saw a group of people doing morning exercise on the square, and he/she walked over...”

Next, please follow the instructions below to complete the retrieval task and answer the corresponding questions:

**Retrieval Task**

Please recall an event that you personally experienced, one that was important to you and brought you strong negative emotions at the time. This event must have occurred within the past year (12 months) but more than one day (24 hours) ago.

Please use the 1^st^-person pronoun “I” to write down this event. You have 10 minutes to recall and write down this event.
