# Supplementary material for: Shifting narrative perspective and construal level shape emotional response and enhance eudaimonic well-being
Source: Sci Rep. 2025 Jun 5;15:19778. doi: 10.1038/s41598-025-01946-8 (PMC12141604; doi:10.1038/s41598-025-01946-8)
Supplement: Supplementary file 2 — Supplementary Material 2 [file 41598_2025_1946_MOESM2_ESM.docx]

**Appendix B: Results on the autobiographical memory characteristics**

**Impacts of Construal Level and Manipulation Relatedness on AM Characteristics**

We focused on the data of Session 2 when participants recalled AM events from the 1^st^-person perspective and fitted 2 (construal level) × 2 (relatedness of construal level manipulation) GLMs. The characteristics of AM events were entered as dependent variables in separate models. In all models, the pre-recall positive and negative affect in Session 2 were entered as covariates. Table B1 presents the means and standard deviations of AM characteristics. Table B2 shows the statistics of these analyses. Only significant results were reported here.

The main effect of construal level was significant on the self-reported material changes brought out by the events: High construal levels brought about fewer changes in material circumstances than low construal levels. The main effect of manipulation relatedness was significant on psychological change of the events: participants rated the AM events to bring more psychological impact when the construal level manipulation was embedded in AM retrieval. The findings suggest that the manipulations of construal level and manipulation relatedness were effective. In contrast to the Construal Level Theory, the effect of construal level on the perceived psychological distance of the events was not significant.

**Impacts of Construal Level, Manipulation Relatedness, and Narrative Perspective on AM Characteristics**

We included Sessions 2 and 3 data to examine the main effect of shifting narrative perspective from 1^st^-person to 3^rd^-person and its associated interactions. We fitted 2 (construal level) × 2 (relatedness of construal level manipulation) × 2 (narrative perspective) repeated-measures GLMs. Each of the AM characteristics was entered as the dependent variable in separate models. The pre-recall positive or negative affect in Sessions 2 and 3 were entered as covariates in all models. Table B3 presents the statistics of these analyses.

The effects of narrative perspective were significant on visual perspective, psychological distance, and imagery vividness. After participants shifted from 1^st^-person to 3^rd^-person perspectives, they rated AM events to be associated more with an observer’s perspective (rather than a field perspective) and trigger less vivid images in mind, which generally replicated previous findings (e.g., Gu & Tse, 2016). Importantly, events narrated from the 3^rd^-person perspective were rated as more psychologically distant than those narrated from the 1^st^-person perspective, supporting that shifting narrative perspective could indirectly manipulate psychological distance. The effect of construal level was significant on the material change brought about by the events, indicating that participants reported more material change for events retrieved at a lower (vs. higher) construal level. Finally, the significant effect of manipulation relatedness on psychological change of events suggested that participants reported more psychological changes when the construal level manipulation was embedded (vs. not embedded) in the AM retrieval task.

Table B1. *Means (standard deviations) of AM characteristics*

|  | Construal level ×  Manipulation relatedness  Psychological distance  (narrative perspective) | Low construal level manipulation not embedded in retrieval task  (N=27) | Low construal level manipulation embedded in retrieval task  (N=25) | High construal level manipulation not embedded in retrieval task  (N=25) | High construal level manipulation embedded in retrieval task  (N=26) |
| --- | --- | --- | --- | --- | --- |
| Emotional intensity | Session 2: 1st-person perspective | 4.67 (1.27) | 4.88 (1.45) | 4.92 (1.12) | 4.69 (1.19) |
|  | Session 3: 3rd-person perspective | 4.00 (1.49) | 3.84 (1.40) | 3.72 (1.72) | 3.81 (1.27) |
| Emotional valence | Session 2: 1st-person perspective | 2.67 (0.96) | 2.52 (1.23) | 2.60 (1.15) | 3.15 (1.35) |
|  | Session 3: 3rd-person perspective | 2.78 (0.80) | 3.44 (1.50) | 3.12 (0.97) | 3.54 (1.14) |
| Visual perspective | Session 2: 1st-person perspective | 2.00 (1.49) | 1.92 (1.08) | 1.80 (1.29) | 2.04 (1.64) |
|  | Session 3: 3rd-person perspective | 5.48 (1.31) | 5.72 (1.14) | 5.92 (1.12) | 5.27 (1.93) |
| Psychological distance | Session 2: 1st-person perspective | 38.07 (29.38) | 34.00 (23.39) | 40.20 (27.44) | 44.23 (25.13) |
|  | Session 3: 3rd-person perspective | 45.26 (25.94) | 46.36 (27.58) | 53.48 (26.03) | 57.73 (18.84) |
| Memory importance | Session 2: 1st-person perspective | 5.48 (1.48) | 5.48 (1.08) | 4.96 (1.54) | 5.46 (1.07) |
|  | Session 3: 3rd-person perspective | 5.07 (1.33) | 4.96 (1.24) | 4.96 (1.62) | 5.23 (1.03) |
| Imagery vividness | Session 2: 1st-person perspective | 5.59 (1.08) | 5.76 (1.05) | 6.16 (0.80) | 6.04 (1.22) |
|  | Session 3: 3rd-person perspective | 4.93 (1.44) | 4.52 (1.36) | 4.80 (1.35) | 4.46 (1.56) |
| Retrieval easiness | Session 2: 1st-person perspective | 4.89 (1.39) | 4.92 (1.26) | 4.56 (1.64) | 5.35 (1.02) |
|  | Session 3: 3rd-person perspective | 4.56 (1.40) | 4.76 (1.36) | 4.92 (1.04) | 5.00 (1.23) |
| Transitional impact: Material | Session 2: 1st-person perspective | 3.37 (1.90) | 3.68 (1.57) | 2.76 (1.67) | 2.88 (1.82) |
|  | Session 3: 3rd-person perspective | 3.07 (1.62) | 3.24 (1.59) | 3.08 (1.63) | 2.46 (1.39) |
| Transitional impact: Psychological | Session 2: 1st-person perspective | 5.33 (1.41) | 5.52 (1.00) | 5.04 (1.31) | 5.77 (0.82) |
|  | Session 3: 3rd-person perspective | 4.85 (1.49) | 5.00 (1.50) | 4.76 (1.33) | 5.31 (1.01) |
| Memory age | Session 2: 1st-person perspective | 107.07 (94.77) | 130.60 (106.27) | 134.88 (126.85) | 155.54 (118.50) |
|  | Session 3: 3rd-person perspective | 109.04 (95.57) | 132.84 (104.84) | 135.32 (125.74) | 165.15 (120.32) |
| Word count | Session 2: 1st-person perspective | 310.52 (102.15) | 317.08 (166.03) | 296.48 (117.77) | 402.04 (131.92) |
|  | Session 3: 3rd-person perspective | 267.67 (70.47) | 269.52 (128.75) | 252.16 (100.37) | 329.46 (120.10) |

Table B2. *The main effects of construal level, manipulation-relatedness, and their interactions on AM characteristics in Session 2*

|  | Construal level | |  | Manipulation relatedness | |  | CL × MR | |
| --- | --- | --- | --- | --- | --- | --- | --- | --- |
|  | *F* | *MS* |  | *F* | *MS* |  | *F* | *MS* |
| Emotional intensity | 0.02 | 0.02 |  | 0.01 | 0.02 |  | 0.91 | 1.38 |
| Emotional valence | 1.45 | 2.05 |  | 1.16 | 1.63 |  | 2.30 | 3.24 |
| Visual perspective | 0.03 | 0.06 |  | 0.001 | 0.002 |  | 0.29 | 0.58 |
| Psychological distance | 1.36 | 951.99 |  | 0.26 | 182.94 |  | 0.67 | 467.05 |
| Memory importance | 2.13 | 3.23 |  | 1.81 | 2.75 |  | 0.97 | 1.48 |
| Imagery vividness | 3.70 | 4.12 |  | 0.004 | 0.01 |  | 0.53 | 0.60 |
| Retrieval easiness | 0.01 | 0.02 |  | 0.73 | 1.30 |  | 1.87 | 3.30 |
| Transitional impact: Material | **4.85*** | **14.67** |  | 0.56 | 1.69 |  | 0.09 | 0.26 |
| Transitional impact: Psychological | 0.13 | 0.16 |  | **5.28*** | **6.68** |  | 1.42 | 1.80 |
| Memory age | 1.18 | 14840.10 |  | 1.45 | 18246.21 |  | 0.004 | 54.02 |
| Word count | 1.93 | 33390.00 |  | 2.84 | 49106.88 |  | 3.55 | 61430.36 |

Note: * *p*<0.05; MS=Mean Square, CL=Construal Level, MR=Manipulation Relatedness; df=(1,97); In Session 2, the narrative perspective was manipulated by the first-person perspective in all groups.

Table B3. *The main effects of construal level, manipulation relatedness, narrative perspective, and their interactions on AM characteristics*

|  | Construal level | |  | Manipulation relatedness | |  | Narrative perspective | |  | CL × MR | |  | CL × NP | |  | NP × MR | |  | CL × MR × NP | |
| --- | --- | --- | --- | --- | --- | --- | --- | --- | --- | --- | --- | --- | --- | --- | --- | --- | --- | --- | --- | --- |
|  | *F* | *MS* |  | *F* | *MS* |  | *F* | *MS* |  | *F* | *MS* |  | *F* | *MS* |  | *F* | *MS* |  | *F* | *MS* |
| Emotional intensity | 0.58 | 1.43 |  | 0.33 | 0.80 |  | **3.80†** | **4.24** |  | 0.03 | 0.07 |  | 0.40 | 0.44 |  | 0.23 | 0.25 |  | 1.45 | 1.61 |
| Emotional valence | 2.53 | 4.72 |  | 3.19 | 5.95 |  | 2.54 | 1.98 |  | 0.29 | 0.55 |  | 0.07 | 0.06 |  | 1.30 | 1.01 |  | 3.53 | 2.75 |
| Visual perspective | 0.001 | 0.002 |  | 0.58 | 1.05 |  | **29.19***** | **63.38** |  | 0.61 | 1.11 |  | 0.001 | 0.001 |  | 0.23 | 0.50 |  | 1.85 | 4.03 |
| Psychological distance | 3.76 | 3994.28 |  | 0.19 | 204.80 |  | **4.23*** | **970.38** |  | 0.35 | 367.51 |  | 0.76 | 173.99 |  | 0.05 | 10.75 |  | 0.38 | 87.61 |
| Memory importance | 0.72 | 1.83 |  | 1.41 | 3.58 |  | 0.01 | 0.01 |  | 0.95 | 2.41 |  | 1.95 | 1.22 |  | 0.60 | 0.37 |  | 0.02 | 0.01 |
| Imagery vividness | 0.69 | 1.21 |  | 0.75 | 1.31 |  | **5.31*** | **7.64** |  | 0.10 | 0.17 |  | 1.35 | 1.94 |  | 0.42 | 0.60 |  | 0.15 | 0.21 |
| Retrieval easiness | 0.09 | 0.21 |  | 0.43 | 1.03 |  | 0.18 | 0.16 |  | 0.60 | 1.43 |  | 0.21 | 0.19 |  | 0.85 | 0.75 |  | 2.14 | 1.89 |
| Transitional impact: Material | **4.81*** | **20.06** |  | 0.05 | 0.21 |  | <0.001 | <0.001 |  | 0.63 | 2.63 |  | **4.00†** | **4.61** |  | 2.42 | 2.78 |  | 1.93 | 2.22 |
| Transitional impact: Psychological | 0.08 | 0.17 |  | **6.63*** | **14.10** |  | 1.90 | 1.52 |  | 1.43 | 3.04 |  | 0.57 | 0.45 |  | 0.001 | <0.001 |  | 0.18 | 0.14 |
| Memory age | 1.53 | 38557.77 |  | 1.93 | 48746.69 |  | 1.36 | 194.02 |  | 0.001 | 35.03 |  | 0.25 | 35.73 |  | 1.82 | 259.45 |  | 2.39 | 340.45 |
| Word count | 1.65 | 43147.17 |  | 2.72 | 71350.71 |  | 3.66 | 11874.68 |  | 3.59 | 93981.85 |  | 0.89 | 2903.78 |  | 0.52 | 1700.37 |  | 0.45 | 1468.08 |

Note: * *p*<0.05, ** *p*<0.01, *** *p*<0.001, † *p*=0.05; *df*=(1, 95); *MS*=Mean Square, NP=Narrative Perspective, CL=Construal Level, MR=Manipulation Relatedness. Across Sessions 2 and 3, the narrative perspective was manipulated as a within-subject variable.
